# Sophisticated Deception in Junior Middle School Students: An ERP Study

**DOI:** 10.3389/fpsyg.2018.02675

**Published:** 2019-01-11

**Authors:** Haizhou Leng, Yanrong Wang, Qian Li, Lizhu Yang, Yan Sun

**Affiliations:** ^1^ School of Psychology, Liaoning Normal University, Dalian, China; ^2^ Xingtai Special Education School, Xingtai, China

**Keywords:** deception, sophisticated deception, cognitive control, ERP, MFN

## Abstract

Sophisticated deception refers to the deception of others based on inferences of their mental states (e.g., answering honestly when inferring that the other will not believe their answer). Studying the brain mechanism of sophisticated deception in junior middle school students can provide physiological evidence for deception detection and deceptive ability measurement. Sixteen junior middle school students were asked to engage in different trial types (i.e., instructed truth/lie and chosen truth/lie), during which we recorded their response times (RT) along with electroencephalographic data to calculate event-related potentials (ERPs). We observed significant differences in amplitude [N2, P3, N450, and medial frontal negativity (MFN)] between chosen reactions (sophisticated deception and simple deception) and instructed reactions (instructed truth and instructed lie) in both the stimulus presentation and feedback stages. In the former, the task scores of participants in the chosen condition were significantly and positively correlated with the N2 amplitude over the central brain area during sophisticated deception. In the latter, the task scores of participants in the chosen condition were negatively correlated with the MFN amplitude over the left frontal and left frontocentral regions. Overall, deception intention, rather than simply making counterfactual statements, appears to underlie the increased demand for cognitive control in deceivers. This can be attributed to deceivers’ need to strongly consider their opponent’s mental state—the better the deceivers’ deceptive ability, the more they will make conjectures about the mental state of their opponent with sophisticated deception and monitor conflict; the less conflict they experience while answering honestly with the intention to deceive, the more conflict may arise when the results of their deception are inconsistent with these conjectures.

## Introduction

Deception refers to the behavior of intentionallymisleading others. Deceptive behavior is important for social communication and is not necessarily an inherently negative event—while some instances of deception are antisocial and selfish in nature, others are prosocial and altruistic in nature, such as jokes or white lies (DePaulo et al., 2003).

Humans use many ways to deceive others: they may intentionally hide information; provide incorrect information; or even tell the truth to others who are predisposed to not believe them, which is called “double bluffing” (Happé, 1994) or sophisticated deception (Sutter, 2009; Volz et al., 2015) or “second-order lying” (Ding et al., 2014; Sai et al., 2018a,b). Sophisticated deception is the higher order behavior of simple deception (statement of counterfactual information). It involves guessing the mental states of others—that is, determining whether the others believe in them—and then choosing appropriate strategies to deceive. When deceivers guess that the other person does not believe in them, they may state the truth in order to successfully deceive. Conversely, when the other person believes in them, they may offer misleading or counterfactual information (commonly known as lying) to achieve their goal.

Thus, when deceiving others, individuals must control both their own and others’ mental states. Lying is considered more cognitively demanding than truth telling (DePaulo et al., 2003; Vrij et al., 2011; Suchotzki et al., 2015). Indeed, event-related potential (ERP) and neuroimaging studies have shown that simple deception requires greater demand for cognitive control than truthful responses; individuals must allocate mental resources to task-related information, inhibit their predominant responses, and resolve response conflict (Botvinick et al., 2001). Lie responses, compared with truthful ones, are associated with increased activation in several prefrontal regions [e.g., dorsolateral prefrontal cortex (PFC) and anterior cingulate cortex (ACC)] linked to cognitive control (Ganis et al., 2003; Nuñez et al., 2005; Abe et al., 2007).

In the past, researchers studied deception using the instructed deception paradigm, whereby participants responded honestly or deceptively based on the instructions they received (Spence et al., 2001; Yokota et al., 2013). However, this paradigm differs from deceptive behavior in real life, meaning that it has low ecological validity. This is because it compels participants to be passive in their deception, rather than to be active. Lewis et al. (1989) used “temptation resistance paradigm” to study deception, which was higher in ecological validity. Some researchers have focused on active deception (Johnson et al., 2003, 2004, 2005; Sun et al., 2011; Ding et al., 2017) and sophisticated deception (Sutter, 2009; Carrión et al., 2010; Ding et al., 2014; Volz et al., 2015; Sai et al., 2018a,b). Some researchers believed that the mere act of suppressing honest responses and making counterfactual statements led to this increased demand for cognitive control (Spence et al., 2001; Ganis et al., 2003; Kozel et al., 2004; Johnson et al., 2005; Nuñez et al., 2005; Abe et al., 2007). Carrión et al. (2010) found that deception intention was the key to the increased demand for cognitive control in deception, using an innovative experimental paradigm. They examined the brain mechanisms of sophisticated deception using a face-to-face interpersonal interaction situation between a “deceiver” and an opponent. The experiment was divided into instructed and chosen conditions: In the former, deceivers had to deceive or respond honestly in response to instructions provided by the computer. In the latter, the deceiver was free to choose whether to respond honestly or deceptively. Polak and Harris (1999) noted that the better a child’s theory of mind (also referred to as “mentalizing”), the more likely they were to engage in deceptive behavior after making a mistake. Therefore, theory of mind likely played an important role in deception (Hala and Russell, 2001). However, Carrión et al. (2010) found that people with better mentalizing ability were less able to deceive. They theorized that the reason for this was that participants with better mentalizing ability experienced more conflict when attempting to deceive. Such conflict in turn interfered with their ability to deceive, such as hindering their ability to control their facial expressions; this meant that they were more likely to expose their true feelings in front of opponents, leading to a lower success rate in deception. This result was counterintuitive, meaning that their results needed to be explored in more depth. This paradigm has its advantages. However, it may also increase the psychological burden of deceiving, particularly if applied in certain cultures—for example, in China, only relatively close individuals can engage in eye contact for extended periods. For this reason, we improved on Carrión et al.’s experimental paradigm to alleviate the psychological burden placed on participants while preserving the paradigm’s ecological validity. Furthermore, to explore their counterintuitive results, we analyzed the feedback stage of their experiment as well.

Researchers used “faux pas recognition” to measure the level of theory of mind of 7- to 11-year-old children, and found that this paradigm could effectively measure their level of theory of mind. If people spoke in an embarrassing and offensive way, or even hurt others, and the speaker did not realize what he should not say, which created faux pas situation (Baron-Cohen et al., 1999). In our research, the Faux Pas Recognition task is also used to measure the level of theory of mind of junior middle school students. Some researchers believed that children’s deception occurred before the age of four (Lewis et al., 1989; Hala et al., 1991), and some other researchers believed that such deception occurred after this age. Only children with theory of mind demonstrated deceptive behavior and had the ability to deceive (Lewis et al., 1990; Sodian et al., 1991; Polak and Harris, 1999). Social and cognitive factors might play an important role in children’s lie-telling abilities (Talwar and Lee, 2008). Children could tell second-order lies by the age of four (Sai et al., 2018a). In the past, there were more studies on children and less on middle school students. Middle school students aged 13–15 are at the second peak period of their physical development, the psychological “weaning period,” and are undergoing the second leap period of self-consciousness. Therefore, it is of great significance to investigate the characteristics of theory of mind among middle school students aged 13–15 (puberty) and their relationship with deception.

## Materials and Methods

### Participants

Participants were recruited from a junior middle school in Dalian. A total of 20 junior middle school students were randomly selected, including 9 males and 11 females. One participant was ultimately excluded because of her low accuracy in the instructed condition, two participants were excluded because they made false responses in the feedback stage, and one participant was excluded because of excessive artifacts in his electroencephalography (EEG) data. Therefore, 16 participants were included in the analysis (aged 12–14 years, M = 13 years, standard deviation = 5 months; 5 boys). All the participants were right-handed, had no history of physical or mental illness, and had normal or corrected visual acuity. None of the participants had participated in a similar experiment before. Their parents/legal guardians signed informed consent before the experiment.

### Procedure

We based our experimental procedure on the paradigm of Carrión et al. (2010). First, all participants were assigned to the “deceiving group.” They faced one opponent (a research assistant) throughout the experiment. The participants sat face-to-face with the opponent; they were obscured from each other’s vision by a set of curtains placed directly between them (made of an ordinary, opaque material with no pattern). The participants were told that they would play a game with their opponent to see who would win and that the higher their scores, the greater their reward.

Both the deceivers and the opponent sat at a desk with a computer in front of them, on which the experimental material was presented (using E-Prime 2.0). The participants sat about 60 cm away from the screen, with their eyes fixed on its center. They were asked to limit their blinking and movement as much as possible.

In the instructed condition, both the deceiver and opponent were simultaneously presented with a fixation cross (“+”) for 500 ms. Then, the deceiver’s experimental procedure began. They were simultaneously presented with indicative signals asking them to be honest or deceptive coupled with original graphics (“△” or “□”). This information remained on the screen for 5,000 ms. These stimuli were visible only to the deceiver. The goal of the deceiver was to deceive the opponent as much as possible during the game, while the opponent’s task was to guess what the original graphics were; in other words, the deceiver had to prevent the opponent from guessing what the graphics were. The deceiver then pressed a key representing each original graphic (“F” = □ and “J” = △) depending on the indicative signal they received. For example, when the participant was given a “deceptive” indicative signal and an original graphic of “△,” he pressed the key corresponding to the “□” graphic (F). The participant had to respond within 5 s. In order to prevent the participant from forgetting what “F” and “J” represented, the graphs of square and triangle were respectively presented in the lower left and lower right of the original graphic. After the participant pressed the key, a voice would state his chosen graphic to the opponent *via* a headset. The vocal response lasted for about 2,000 ms. After 2,000 ms, the opponent could respond, and the words “waiting for reply” appeared on the participant’s screen. For example, if the participant pressed the square key (F), the opponent heard “square” through their headset. The opponent needed to judge whether the deceiver told them the correct answer, guessed the correct answer and pressed the corresponding key. The opponent’s response was subsequently presented (in the form of graphic) on the deceiver’s computer screen for 2,000 ms. The deceiver then indicated whether the opponent was correct or not by pressing the right key “F” or the wrong key “J.” To prevent the participants from forgetting the original graphics, they were presented on the screen when participants made the judgment. If the opponent guessed correctly, they won the match and the deceiver lost three points. However, if the opponent guessed incorrectly, the deceiver gained three points. The current score and running total score were presented to the deceiver and opponent for 1,500 ms after the feedback.

**Figure 1:**
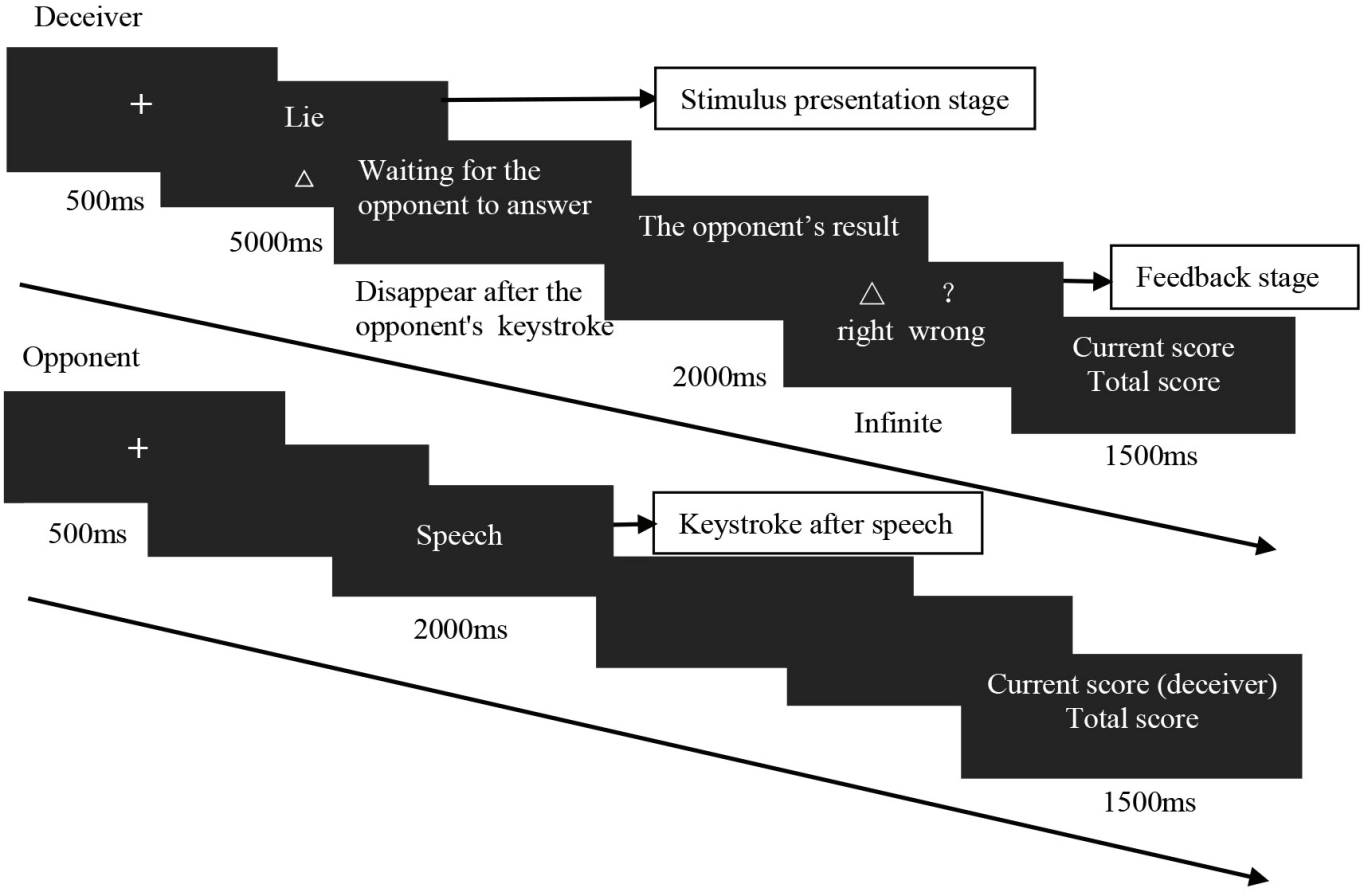
Experimental flowchart.

The instructed condition contained two blocks. Each block comprised 30 trials, for a total of 60. See Figure 1 for the specific experimental procedure.

The procedure of the chosen condition was the same as that of the instructed condition. The only difference was that the deceiver was initially presented with the word “select” (instead of the instruction on whether to deceive or be honest), prompting the deceiver to choose whether to deceive or respond honestly. Because the opponent would not know how the deceiver responded (i.e., honestly or deceptively), the deceiver had to adopt a strategy in deciding whether to deceive or respond honestly. Again, their goal was to make the opponent guess incorrectly. For example, after successfully deceiving an opponent for the first time, the deceiver might believe that the opponent would not choose to believe the deceiver. Accordingly, the deceiver could deliberately not deceive the opponent so that the opponent again made the wrong decision. The chosen condition contained 3 blocks, each comprising 34 trials (for a total of 102 trials). To ensure that the participants answered carefully, participants were given a gift that was aligned with their final score. The participants first completed the experiment of the instructed condition and then completed the experiment of the chosen condition. All the participants faced the same opponent.

Following the experiment, we used the Faux Pas Recognition task and eyes task (Bai, 2011) to measure the level of theory of mind of middle school students. The Faux Pas Recognition task came from Baron-Cohen et al. (1999), and there were two tasks. Each task has been translated and modified in detail according to the Chinese cultural background, and the task context was adapted for the understanding and measurement of middle school students in China. Baron-Cohen designed and revised the eyes task (Baron-Cohen et al., 1997; Baron-Cohen et al., 2001), which involved pairing mental states with emotional expressions. Bai (2011) translated it into Chinese.

### Statistical Analysis

An electrode cap (Brain Products GmbH) was used to collect the EEG data. The cap covered 64 scalp sites with tin electrodes, using a sampling frequency of 500 Hz (impedance <5 kΩ). Vertical electrooculograms (VEOG) were recorded simultaneously to monitor eye movements. The ERP waveforms were then re-referenced offline to the average of the left and right mastoids. We averaged the ERPs of each condition and applied digital filtering with a low-pass, half-power cut-off frequency of 30 Hz. For each trial, channels were marked as artifacts if the signal variation exceeded ±100 μV. In the stimulus presentation stage, we examined two ERP components—the N2 (time window = 240–320 ms) and N450 (time window = 400–500 ms)—at electrode sites F7, F5, F3, F1, Fz, F2, F4, F6, F8, C5, C3, C1, Cz, C2, C4, C6, Fc1, Fc2, Fc3, Fcz, Fc4, Fc5, and Fc6. Furthermore, we identified the P3 component (time window = 320–400 ms) at electrode sites CP5, CP3, CP1, CPz, CP2, CP4, CP6, P7, P5, P3, P1, Pz, P2, P4, P6, and P8. The analysis schedule was conducted to stimulate the pictures from before 200 to 1,000 ms after the stimulus pictures appeared, to stimulate the pictures before 200 ms as a baseline. In the feedback stage, we identified the medial frontal negativity (MFN) component (time window = 240–340 ms) at electrode sites F7, F5, F3, F1, Fz, F2, F4, F6, F8, C5, C3, C1, Cz, C2, C4, C6, Fc1, Fc2, Fc3, Fcz, Fc4, Fc5, and Fc6. We also identified the P3 component (time window = 340–500 ms) at electrode sites CP5, CP3, CP1, CPz, CP2, CP4, CP6, P7, P5, P3, P1, Pz, P2, P4, P6, and P8. The mean amplitude method was used for statistical analysis. For the analyses of variance (ANOVAs), *p* were corrected *via* the Greenhouse-Geisser method.

## Results

### Behavioral Results

Two participants were excluded because they pressed the buttons at random during the feedback stage of the experiment. The accuracy rate of the participants who were involved in the statistical analysis in the instructed condition was over 88%, whereas the accuracy rate of one participant was 65%; this suggested distraction, and thus they were excluded from the experiment. The mean reaction time and standard deviation of the participants in the four conditions are shown in Table 1.

**Table 1:** Mean reaction time and standard deviation (ms) of participants in the four conditions.

Trial type	M	SD
Instructed truth	1360.774	60.632
Instructed lie	1530.427	90.417
Chosen truth	1510.372	115.807
Chosen lie	1497.785	116.214

The mean reaction times for the four experimental types were analyzed *via* two-way repeated measures analysis of variance [2 (condition: instructed, chosen) × 2 (strategy: truth, lie)]. The results revealed that the main effect of condition was not significant [F(1,15) = 0.296, *p* = 0.594, and $\eta_{p}^{2}$ = 0.105], and neither was the main effect of strategy [F(1,15) = 3.852, *p* = 0.069, and $\eta_{p}^{2}$ = 0.204]. However, the interaction between condition and strategy was significant [F(1,15) = 12.066, *p* = 0.003 < 0.05, and $\eta_{p}^{2}$ = 0.446]. Further tests revealed that the reaction time for instructed truth was significantly lower than that for instructed lie [t(15) = −3.735 and *p* = 0.002], but there was no significant difference between chosen truth and chosen lie [t(15) = −0.251 and *p* > 0.05].

Under the chosen condition, the average number of times of chosen truth by the participants was 44.312 (SD = 5.186), and the average number of times of chosen lie was 57.687 (SD = 5.186). The paired sample t-test revealed that there was a significant difference between the number of times of chosen truth and chosen lie [t(15) = −5.158 and *p* < 0.001]. The number of times of chosen lie was more than that of chosen truth. Under the chosen condition, the average scoring rate (percentage of scoring times) selected by the participants was 0.529 (SD = 0.044). We used the scoring rate to reflect the participant’s ability to cheat. The average score of the Faux Pas Recognition task was 18.625 (SD = 5.301), and the average score of the eyes task was 8.500 (SD = 2.251).

Correlation analyses revealed that there was no significant correlation between the scores in the Faux Pas Recognition task and the scoring rate (r = 0.194 and *p* > 0.05) and the number of times of chosen truth (r = 0.052 and *p* > 0.05). Correlation analyses showed that there was no significant correlation between the scores on the eyes task and the scoring rate (r = 0.052 and *p* > 0.05) and the number of times of chosen truth (r = 0.178 and *p* > 0.05). There were significant positive correlations between the scores of the Faux Pas Recognition task and the eyes task (r = 0.600 and *p* < 0.05).

### ERP Results

#### ERP Waveform Analysis in the Stimulus Presentation Stage

**Figure 2:**
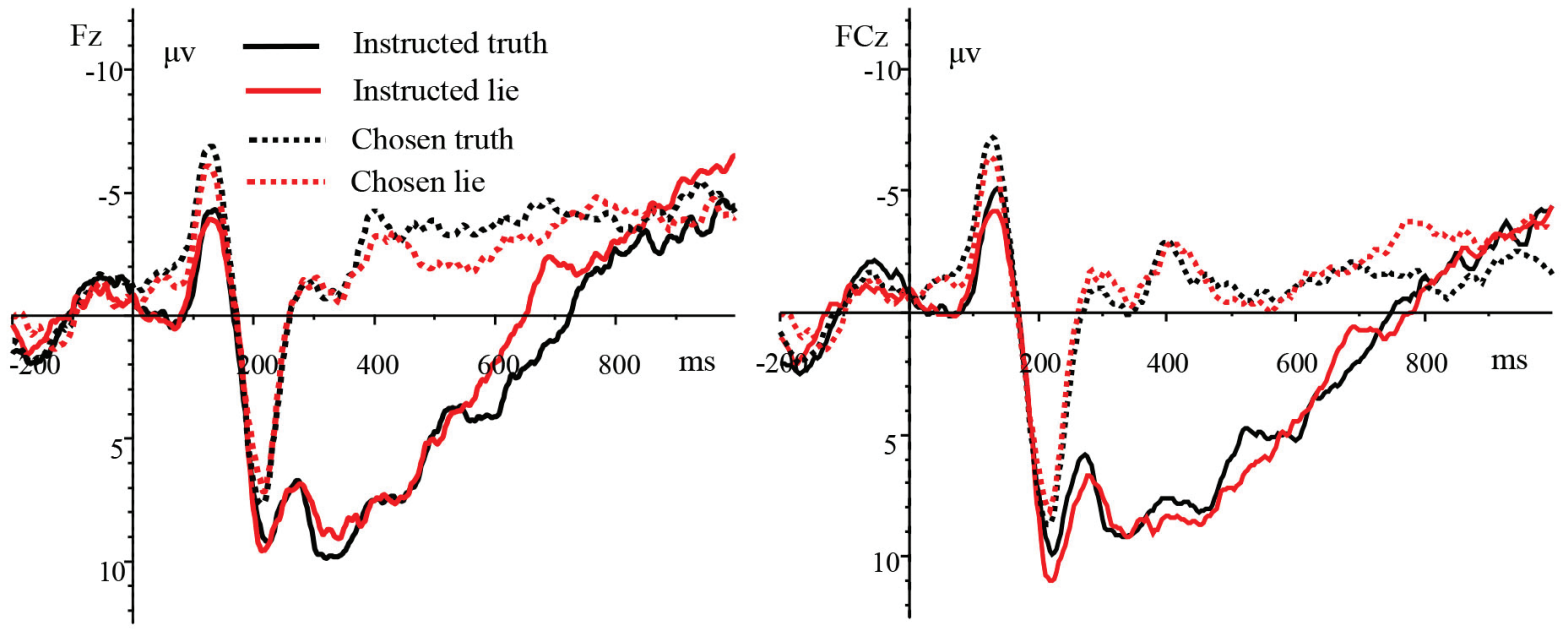
Grand-average ERPs of the four trial types at electrode sites Fz and FCz.

The analysis of ERP waveforms after stimulus presentation showed that the N2 (240–320 ms) component appeared over the frontal, frontocentral, and central regions in both the instructed and chosen conditions; the P3 (320–400 ms) appeared over the parietal and centroparietal regions in the instructed condition; and the N450 (400–500 ms) appeared over the frontal, frontocentral, and central regions in the chosen condition. The grand-average ERPs of the four experimental types at electrode sites Fz and FCz are shown in Figure 2.

We then analyzed the average amplitude of the N2 using a three-way repeated measures ANOVA [4 (trial type: instructed truth, instructed lie, chosen truth, chosen lie) × 3 (laterality: left, midline, right) × 3 (brain area: frontal, frontocentral, central)]. The results revealed a significant main effect of trial type [F(3,45) = 14.703, *p* < 0.001, and $\eta_{p}^{2}$ = 0.495] but non-significant main effects of laterality [F(2,30) = 0.924, *p* = 0.362, and $\eta_{p}^{2}$ = 0.058] and brain area [F(2,30) = 1.319, *p* = 0.273, and $\eta_{p}^{2}$ = 0.081]. The interaction between trial type and laterality was significant [F(6,90) = 4.283, *p* < 0.01, and $\eta_{p}^{2}$ = 0.222], while the interaction between trial type and brain area was not significant [F(6,90) = 1.773, *p* = 0.163, and $\eta_{p}^{2}$ = 0.106]. The interaction between laterality and brain area was not significant [F(4,60) = 2.272, *p* = 0.072, and $\eta_{p}^{2}$ = 0.132]. Finally, the interaction between the experimental type, brain area, and laterality was significant [F(12,180) = 2.559, *p* < 0.05, and $\eta_{p}^{2}$ = 0.146]. Further tests indicated that the chosen truth and chosen lie induced a more negative N2 compared to the instructed lie and instructed truth. There was no significant difference in N2 amplitude between chosen truth and chosen lie or between instructed lie and instructed truth. Correlation analyses between participants’ scores and the N2 amplitude found that there were significant positive correlations between the score and the N2 amplitude over the midline region in the chosen truth condition [r(Fz) = 0.500, r(Fcz) = 0.523, r(Cz) = 0.524, and *p* < 0.05].

We then subjected the average amplitude of the P3 to a three-way repeated measures ANOVA [2 (trial type: instructed truth, instructed lie) × 3 (laterality: left, midline, right) × 2 (brain area: parietal, centroparietal)]. None of the main effects or interactions was significant; that is, there was no significant difference in P3 amplitude in instructed truth and instructed lie over the parietal and centroparietal scalps. A similar analysis of the N450 using a three-way repeated measures ANOVA [(trial type: chosen truth, chosen lie) × 3 (laterality: left, midline, right) × 3 (brain area: frontal, frontocentral, central)] also revealed no significant main effects or interactions—the N450 amplitude did not differ between chosen truth and chosen lie over the frontal, frontocentral, and central scalp regions.

#### ERP Waveform Analysis in the Feedback Stage

**Figure 3:**
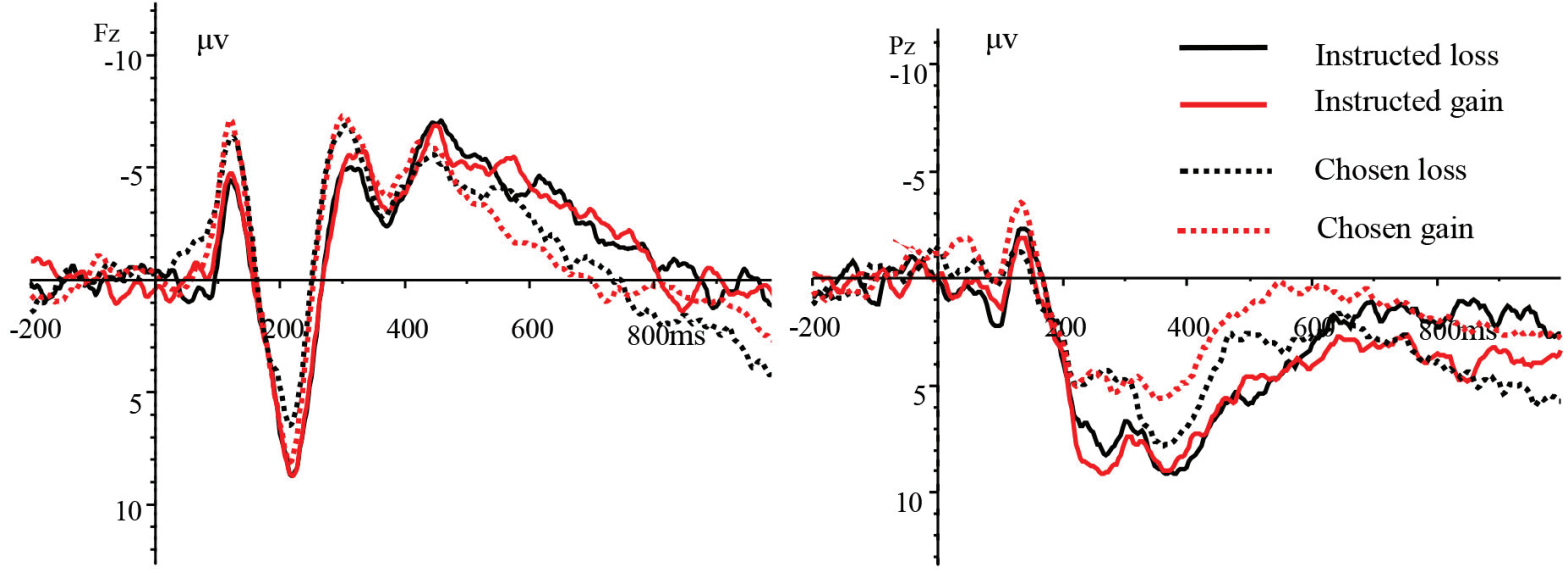
Grand-average ERPs of the four scoring types at electrode sites Fz and Pz.

The EEG analysis during the feedback stage (when the feedback keys were presented) revealed the MFN (240–340 ms) over the frontal, frontocentral, and central scalp regions and the P3(340–500 ms) over the parietal and centroparietal scalp regions. The feedback results could be divided into four types based on scoring: instructed loss, instructed gain, chosen loss, and chosen gain. The grand-average ERPs of the four scoring types at electrode sites Fz and Pz are shown in Figure 3.

The average amplitude of the MFN was subjected to a three-way repeated measures ANOVA [4(scoring type: instructed loss, instructed gain, chosen loss, and chosen gain) × 3 (laterality: left, midline, right) × 3 (brain area: frontal, frontocentral, and central)]. The results revealed non-significant main effects of scoring type [F(3,45) = 1.529, *p* = 0.236, and $\eta_{p}^{2}$ = 0.092] and laterality [F(2,30) = 0.891, *p* = 0.421, and $\eta_{p}^{2}$ = 0.056], but a significant main effect of brain area [F(2,30) = 64.031, *p* < 0.001, and $\eta_{p}^{2}$ = 0.810]. The interaction between scoring type and laterality was also significant [F(6,90) = 14.390, *p* < 0.001, and $\eta_{p}^{2}$ = 0.490]. However, the interaction between scoring type and brain area was not significant [F(6,90) = 0.787, *p* = 0.499, and $\eta_{p}^{2}$ = 0.050] nor was the interaction of laterality and brain area [F(4,60) = 1.309, *p* = 0.285, and $\eta_{p}^{2}$ = 0.080]. The three-way interaction of scoring type, brain area, and laterality was significant [F(12,180) = 5.112, *p* < 0.01, and $\eta_{p}^{2}$ = 0.254]. Further tests revealed that in the left hemisphere, the MFN amplitude was more negative for the instructed gain type than for the instructed loss type, for the chosen gain type than for the chosen loss type, and for the chosen gain type than for the instructed loss type. Furthermore, in the right hemisphere, the amplitude was more negative for the instructed loss type than for the instructed gain type, for the chosen loss type than for the chosen gain type, and for the chosen loss type than for the instructed gain type. Correlation analyses showed that there was a significant negative correlation between the score in the chosen condition and the MFN amplitude over the left side of the frontal scalp region (r = −0.595 and *p* < 0.05) and the MFN amplitude over the left side of the frontocentral scalp for the chosen loss type (r = −0.545, *p* < 0.05).

The P3 amplitude was also subjected to a three-way repeated measures ANOVA [4 (scoring type: instructed loss, instructed gain, chosen loss, chosen gain) × 3 (laterality: left, midline, right) × 2 (brain area: parietal, centroparietal)]. We found a non-significant main effect of scoring type [F(3,45) = 2.801, *p* = 0.073, and $\eta_{p}^{2}$ = 0.157], but a significant main effect of laterality [F(2,30) = 3.694, *p* < 0.05, and $\eta_{p}^{2}$ = 0.198]. The main effect of brain area was not significant [F(1,15) = 1.535, *p* = 0.234, and $\eta_{p}^{2}$ = 0.093]. The interaction between scoring type and laterality was also significant [F(6,90) = 3.668, *p* < 0.01, and $\eta_{p}^{2}$ = 0.196], while the interactions between scoring type and brain area [F(3,45) = 1.877, *p* = 0.147, and $\eta_{p}^{2}$ = 0.111] and between laterality and brain area were not significant [F(2,30) = 0.903, *p* = 0.416, and $\eta_{p}^{2}$ = 0.057]. The three-way interaction between scoring type, brain area, and laterality was not significant [F(6,90) = 2.413, *p* = 0.076, and $\eta_{p}^{2}$ = 0.140]. Further tests revealed that in the left hemisphere, the P3 amplitude was more positive for the instructed loss type than for the instructed gain type and for the instructed loss type than for the chosen gain type. In the right hemisphere, the P3 amplitude was more positive for the instructed gain type than for the chosen gain type and for the instructed gain type than for the chosen loss type.

## Discussion

We found that in the instructed condition, it took longer to engage in lying than in telling the truth, whereas in the chosen condition, there was no significant difference in response time (RT) between telling a lie or a truth. These findings were consistent with previous studies (Spence et al., 2004; Carrión et al., 2010). An instructed lie required a counterfactual statement, which naturally made it take longer than an instructed truth. The lack of RT difference in the chosen condition was likely because the chosen response required the deceiver to consider the mental state of the opponent, regardless of whether it involved a contrary statement or not.

For successful deception, a major requirement is mentalizing (Frith and Frith, 2003, 2006; Sip et al., 2008). Lie responses are associated with increased activation in several prefrontal regions (Ganis et al., 2003; Nuñez et al., 2005; Abe et al., 2007). Inhibition of the anterior PFC can improve deceptive behavior (Karim et al., 2009). Reasoning about others’ mental states mainly activates the ACC, PFC, and the temporoparietal junction (TPJ) (Krippl and Karim, 2011; Corradi-Dell’Acqua, 2013; Daltrozzo et al., 2016). Two brain systems (the mirror and the mentalizing systems) are thought to be involved in the processing of understanding intention (Ciaramidaro et al., 2014). Khalil et al. (2018) suggested a multilayer neural network model including the mirror neuron system (MNS) on a first layer and transforming this information to a higher layer network responsible for reasoning. ERP studies of intention are quite limited. Carrión et al. (2010) found that the N450 was related to deception intention and people with better mentalizing ability were less able to deceive. Our results are different from Carrión et al.’s, there was no significant correlation between the mentalizing ability and the ability to cheat and the tendency of chosen truth. We think that it is probably because sophisticated deception is more difficult, and the mentalizing ability of middle school students is also developing.

In the stimulus presentation stage, a more negative N2 was induced for chosen truth or lie compared to instructed lie or truth. Both the amplitude and latency of the N2 related to the state of the individual performing the task, and the N2 generally arose in conflict control tasks. When individuals could quickly identify conflicting information and began the correct processing pathway, the latency of the N2 was generally short and the amplitude was small. By contrast, when individuals had to deal with complex tasks, the N2 amplitude became more negative and the latency lengthened, leading to greater brain activity (Azizian et al., 2006; Folstein and Van Petten, 2008). In the instructed condition, participants only had to press the indicated button, rather than deciding on their own, so it was easier for them to deal with conflicting information than in the chosen condition. First, participants made an inference about the opponent’s mental state, and then decided whether the next stimulus was honest or lying reaction. Honest or lying reaction is not decided by participants, and the instructions are given by the computer. It may or may not be consistent with the idea of the participants. The final score was unrelated to the participants, because this is not the active behavior of the participants, but the points lost and scored as a result of computer manipulation. The participants were not responsible for losing points and scoring points. Later, the participants no longer considered the mental state of the opponent, and they simply followed the instructions of the computer to respond. In other words, the instructed condition required less cognitive control and conflict processing resources than when engaging in deception (whether sophisticated or simple) in the chosen condition. Thus, the chosen condition naturally induced a more negative N2. In the future, we will use more participants to verify the stability of our results.

Some researchers have also found that instructed or voluntary deception induced a more negative N2 than honesty (Wu et al., 2009; Hu et al., 2011, 2013, 2015; Suchotzki et al., 2015; Ganis et al., 2016). However, a notable difference in our study was that the chosen truth and chosen lie conditions did not differ in terms of N2 amplitude. Similarly, there was no significant difference in N2 amplitude between the instructed lie and truth conditions. This suggested that a deception intention is the key to increasing cognitive control needs, rather than merely making counterfactual statements. The chosen truth and chosen lie had similar intentions and cognitive control processing, which likely explained why there was no difference in N2 amplitude between these conditions. In the instructed condition, even if counterfactual statements were required, participants may not have a strong desire to participate. By contrast, in the chosen condition, whether they chose to be honest or deceive, they likely had an intention to deceive (given that it is essential to completing the task). Sai et al. (2018b) also found that telling a lie or a truth to deceive elicited a larger N200 than honest responses, it is the deceptive intention that elicits response conflict. Participants’ scoring was also significantly and positively correlated with the N2 amplitude over the central brain area in the chosen truth condition (i.e., sophisticated deception). That is, the better the deceptive ability of the participants was, the less conflict they experienced while speculating about the mental state of their opponent to engage in sophisticated deception.

We found that the instructed condition induced the P3, while the chosen condition induced the N450. Furthermore, there was no significant difference in P3 amplitude between the instructed truth and instructed lie conditions, and no difference in N450 amplitude between the chosen truth and chosen lie conditions. The N450 was sensitive to cognitive control needs and conflict processing and was mainly distributed across the ACC and dorsolateral PFC, the former of which was located on the inner surface of the frontal region (West et al., 2004, 2005). During the chosen condition, participants must speculate about the mental state of their opponent, while in the instructed condition, they needed only distinguish between the different instructions to react; these different qualities likely explained why the instructed condition induced the P3 and the chosen condition induced the N450. Carrión et al. (2010) also studied the brain mechanisms of the intention underlying sophisticated deception using a combination of instructed and chosen conditions. They found that the chosen truth or lie and the instructed lie induced a more negative N450 than did the instructed truth, and we agreed with their explanation of this finding as that the N450 was sensitive to deceptive intention. However, their study utilized a face-to-face situation, where deception in the instructed condition would be influenced by the face of the other person, instructed lie generated cognitive conflict much like that of real (i.e., chosen) deception. Although participants faced a real opponent in this study, the opponent was kept out of sight by a curtain, so the instructed lie became a counterfactual statement only as a result of the instructions. Nevertheless, both the instructed lie and instructed truth had less deceptive intention than the chosen truth and lie.

In the feedback stage, both the instructed and chosen loss types induced a more negative MFN over the right hemisphere than did the instructed and chosen gain types, respectively. In the left hemisphere, the opposite pattern was observed. Gehring and Willoughby (2002) named the negative wave that occurred about 200 ms after presenting feedback information in a gambling task the MFN, which corresponded to our results. Furthermore, a loss in their study led to a more negative MFN than did a gain, and the magnitude was positively correlated with gambling risk. In this study, participants’ score was significantly and negatively correlated with the amplitude of the MFN over the left hemisphere of the frontal and central frontal scalp regions in the chosen loss type. These results suggested that the better the participant’s ability to deceive, the more conflict they experienced when the results were inconsistent with what they expected. Carrión et al. (2010) found a counterintuitive result that people with better mentalizing ability were less able to deceive. Our results can offer a possible explanation for this result. Participants with better mentalizing ability pay less attention to the score and experience more conflict when attempting to deceive. Such conflict and less desire to deceive others in turn interfere with their ability to deceive, leading to a lower success rate in deception.

As for the P3 component in the feedback stage, the instructed loss induced a more positive P3 over the left hemisphere than did the instructed or chosen gain. Over the right hemisphere, the instructed gain induced a more positive P3 component than did the chosen gain or chosen loss. P3 was known to relate to attentive resources. Johnson et al. (2004) also proposed that the amplitude of P3 was negatively correlated with the difficulty of the tasks. Taken together, our results indicated that participants paid more attention to the feedback input in the chosen condition than that in the indication condition.

In the past, computers were commonly used as the main opponent in deception research. However, deceptive behavior in interpersonal interaction might involve more social cognitive processing (Ding et al., 2013). Some researchers believed that deception in interpersonal interactions comprised three stages: decision-making, mentalizing, and response inhibition. In the decision-making stage, deceivers must evaluate the risk of deceiving and budget their reward. In the mentalizing stage, the deceiver must repeatedly analyze the thoughts of the other person at that time, and constantly build a reputation for being trustworthy in front of the deceived. Finally, in the response inhibition stage, the deceiver must control his or her real thoughts and behaviors to ensure that the deception proceeded smoothly (Sip et al., 2008). The greatest difficulty in successfully deceiving others was cognitive control. Individuals must not only control their own mental states but also understand the mental states of others, both of which required considerable cognitive control (Blakemore et al., 2004).

Studying the brain mechanisms of sophisticated deception in junior middle school students can provide us with physiological evidence for the measurement of deception detection and deceptive ability. In the past, researchers believed that untruthful statements by nature required more cognitive control. However, we found that deceptive intentions were more critical. In other words, choosing to answer honestly while intending to deceive has a similar demand for cognitive control as simple deception. By contrast, deception in the instructed condition was more reminiscent of inhibitory control, and required no deceptive intention. Thus, the results of studies on stereotyped deceptive behaviors in the laboratory and freely selected deceptive behaviors using a paradigm with good ecological validity differ markedly (Hauch et al., 2015; Levine and Schweitzer, 2015). Unusual intentional behaviors were known to activate the medial frontal cortex more than do stereotypical behaviors in the laboratory (Desmet and Brass, 2015). Deceptive behavior cannot occur free of social context. Jupe et al. (2016) studied the deception detection abilities of teenage offenders and teenage non-offenders in a video deception paradigm, and found that offenders were more accurate in their decisions. This was likely because offenders were more familiar with deception situations, which improved their own ability to deceive.

## Conclusion

In this study, we investigated the brain mechanisms of sophisticated deception in junior middle school students using ERPs. We found that deception intention was the key to the increased demand for cognitive control involved in such deception, as opposed to making counterfactual statements. Furthermore, the better the participants’ ability to deceive, the less conflict they experienced while speculating about the mental state of their opponent when answering honestly with the intention to deceive. Furthermore, the more they monitored conflict during such deception, the more was the conflict they experienced when the results were inconsistent with their expectations.

## Ethics Statement

This study was approved by the local ethics committees of Liaoning Normal University. Written informed consent had been obtained from the parents/legal guardians of all participants.

## Author Contributions

YS conceived this study. HL participated in writing the manuscript. QL participated in reviewing the literature. YW participated in performing the study. LY participated in modifying the manuscript.

### Conflict of Interest Statement

The authors declare that the research was conducted in the absence of any commercial or financial relationships that could be construed as a potential conflict of interest.
